# Effects of Fructus Akebiae on learning and memory impairment in a scopolamine-induced animal model of dementia

**DOI:** 10.3892/etm.2014.1775

**Published:** 2014-06-11

**Authors:** JINGHUA WANG, XUAN WANG, BAOSHENG LV, WEIXIU YUAN, ZEGUO FENG, WEIDONG MI, HONG ZHANG

**Affiliations:** 1 Anesthesia and Operation Center, Chinese PLA General Hospital and Medical School of Chinese PLA, Beijing 100853, P.R. China; 2 Department of Psychiatry, Beijing Huilongguan Hospital, Beijing 100096, P.R. China

**Keywords:** Fructus Akebiae, scopolamine-induced, learning and memory impairment, cognition

## Abstract

Fructus Akebiae (FAE) is a component of traditional Chinese medicines used for the clinical treatment of amnesia. The aim of the present study was to investigate the effects of FAE extract on scopolamine-induced learning and memory impairment in mice and Sprague-Dawley rats. Treatment with FAE (2.5, 5 and 10 mg/kg) was investigated in scopolamine-treated animals, and its effects on different types of memory were examined using the T-maze, the Morris water maze task, the novel object recognition test, the passive avoidance task and the step-down test. The results revealed that 5 and 10 mg/kg FAE attenuated scopolamine-mediated impairment of cognition, including spatial, episodic, aversive, and short- and long-term memory. Overall, these results suggest that FAE is an effective cognitive enhancer, and thus highlights the value of a multi-target strategy to address the complexity of cognitive dysfunction in Alzheimer’s disease.

## Introduction

Alzheimer’s disease (AD) is an age-associated neurodegenerative disorder characterized by progressive loss of memory and cognition, with mortality occurring approximately a decade following diagnosis (1). During the pathogenesis of AD, patients exhibit a slow progression from spatial/episodic memory dysfunction, to a complete decline of cognitive function, at which point they are dependent on caregivers. Presently, there is no cure for AD, and current treatments only offer limited symptomatic benefit (2).

One pharmacological strategy for AD is the use of acetylcholinesterase inhibitors (AChEIs), which inhibit the breakdown of acetylcholine (ACh), and therefore increase its concentration in neuronal synapses to compensate for the loss of cholinergic neurons, which is characteristic of AD (2). Despite the reported benefits of AChEIs for AD, their use is limited since they have severe side-effects (3). Therefore, a greater understanding of the underlying pathophysiological mechanisms may help identify more effective and innovative treatment strategies, and thus the development of novel drugs.

Chinese herbal medicines have been widely used for thousands of years in China and other Asian countries. In the clinical practice of traditional Chinese medicine (TCM), the modification of an original formulation by the addition or substitution of herbs according to the condition of the patient is common to enhance the efficacy of the original formulation (4). The majority of TCMs have not resulted in any serious side-effects. Furthermore, since the mechanism of action involves multiple components, pathways and targets, TCMs may have significant advantages compared with single-component drugs for the treatment of multifactor, complex chronic diseases, including AD (5). Specific TCM formulations have been shown to be effective against numerous cognitive disorders. For example, Fructus Akebiae (*Akebia* fruit; FAE), the fruit of a plant widely distributed throughout China, has been used for the treatment of mental disorders. A number of studies have used FAE as the major ingredient in complex prescriptions for the treatment of mental disorders and cognitive and behavioral deficits, including insomnia, memory loss, paraphasia, phobia and depression (6). A previous study identified that the genus *Akebia* contains more than thirty types of triterpenoid saponins, and the majority of these compounds are derivatives of the triterpenoid hederagenin (7). Several studies have focused on the biological activity of FAE, particularly on its antidepressant-like activity. However, little is known about its effects on cognition and memory *in vitro* and *in vivo* (6).

In animal studies, the muscarinic cholinergic receptor antagonist, scopolamine, has been widely used to induce partial amnesia as a model for dementia (8,9). Therefore, the present study aimed to investigate the effect of FAE on learning and memory impairment in scopolamine-induced mouse and rat models of dementia via tests including the Morris water maze (MWM) task for mice and novel object recognition test for rats.

## Materials and methods

### Preparation of FAE

The fat was removed from FAE dry fruit powder (500 g) twice by ultrasonication in petroleum ether at room temperature, as previously described (10). Fructus Akebiae was obtained from Beijing Tongrentang Medicine Facility, Beijing, China (batch #: 701001532-1). The plant was harvested from Jiangsu Province, China. The solvent was volatilized, and the coarse powder was extracted twice by recirculation for 1 h with 2 l 80% ethanol (11). The extract (500 ml) was concentrated under reduced pressure and then stored overnight at room temperature followed by filtration. As previously described by Wain *et al* (12), water was added to the filtrate, which was then extracted three times with 800 ml ethyl acetate and four times with 800 ml water-saturated n-butanol (n-BuOH). n-BuOH was recycled under reduced pressure and saponins were obtained. A total of 5 g of the saponins was degraded for 3 h with 60 ml HCl (2 mol/l) in 45% ethanol (EtOH) at 100°C. Following filtration and further washes with water to remove the acid, the degraded saponin was dried via vacuum, resulting in crude crystals. These crystals were then dissolved in 300 ml hot 70% EtOH and decolored by heating-recirculation with 1 g activated carbon for 30 min, followed by filtration at 50°C. The filtrate was concentrated for 12 h at 4°C under reduced pressure to obtain clustered crystals. These crystals were then filtered, and chloride ion was removed via washing with water, followed by drying in a vacuum to form a white powder. The yield of the final extract was ~0.5% (w/w). The powder was stored at 4°C until use.

### Animals

Sprague-Dawley rats and ICR mice were provided by the Animal Breeding Center affiliated with Beijing Institute of Pharmacology (Beijing, China). The animals were kept in polyacrylic cages (6 mice per cage; 1 rat per cage) and maintained under standard housing conditions (24–27°C and 60–65% humidity) on a 12 h light:dark cycle, with food (dry pellets) and water available *ad libitum*. However, food was not allowed during the experiment. The use and care of animals was followed according to the guide for laboratory animals of the National Institutes of Health (2010, Bethesda, MD, USA).

### Materials

Scopolamine hydrobromide (Sigma Aldrich, Milan, Italy) was dissolved in 0.9% saline and administered intraperitoneally (i.p.) to mice and rats 10 min prior to testing at 1 or 10 mg/kg. The FAE extracts (13) were suspended in 5% EtOH (Sigma-Aldrich) in 95% water for oral administration (p.o.) at 2.5, 5, or 10 mg/kg (n=6 animals per treatment), and administered 40 min prior to behavioral tests. All other reagents were purchased from Beijing Chemical Reagent Co., Ltd. (Beijing, China).

### MWM task

The MWM task was performed for the assessment of spatial learning and memory, as previously described (14), with some modifications. Briefly, a white circular tank (130 cm diameter and 50 cm high) with a featureless inner surface was used. The pool was filled with opaque water (23±2°C) to a depth of 30 cm. The target platform (10 cm diameter and 31 cm high) was submerged so that 1 cm of the platform was above the water surface. The platform remained in a fixed position at the midpoint of one quadrant throughout the training phase. Training consisted of two daily sessions of four consecutive 60-sec trials, each with a 15-sec inter-trial interval, during which mice were placed in the pool facing the wall, from various starting points and allowed to swim freely to the escape platform. If mice failed to find the platform within the allocated 60 sec, they were guided to the platform by the experimenter. The trial ended as soon as the animal climbed on the platform and remained on it for ≥2 sec. Following each session, each mouse was allowed to remain on the platform for 20 sec prior to being placed in a heated chamber. To accelerate the training, an extra trial was added prior to the first session, in which mice were placed on the hidden platform for 60 sec. Animals were trained until they were able to reach the escape platform in <20 sec. In the single probe trial (performed 24 h following the last training session), the platform was removed and each mouse was allowed to swim in the maze for 60 sec while their behavior was recorded by an automated activity monitoring system (ANY-maze video tracking; Stoelting Co., Wood Dale, IL, USA) and the percentage of time spent in the platform area was calculated. FAE and scopolamine treatments (15) were administered 40 and 20 min, respectively, prior to the first trial (T1) and the probe trial.

### Novel object recognition test

Male Sprague-Dawley rats were housed in the experimental room and regularly handled for 1 week prior to this experiment to test for episodic memory. The test apparatus, a black Perspex box with sawdust bedding, (70×50×40 cm) was indirectly illuminated with a 50 W halogen lamp. Metal triangular (8.5×5×14 cm) and rectangular prisms (5×5×14 cm) were used as objects to be discriminated. On day 1, rats were allowed to explore the test apparatus without objects for 150 sec. Two identical objects were then introduced after 24 h for exploration (sample trial, T1). Object exploration was determined when the rat sniffed and/or touched the object <2 cm from its nose, and when the two objects were explored for ≥10 sec, the sample trial was terminated by moving the rat to its home cage. Retrieval was examined 24 h following T1 (for example, choice trial, T2), when rats were allowed to explore a novel and familiar object for 4 min. The two objects were randomly placed to reduce the potential effect of place or object preference. FAE or vehicle was administered 40 min prior to T1. The behavioral characteristics and exploration time was videotaped and measured, respectively. The total amount of time spent exploring the novel object (N) and the familiar object (F) was used to calculate the discrimination index (N-F/N+F) in T2. Each acquisition trial was performed 10 min following the administration of a single scopolamine treatment (2 mg/kg, i.p.).

### Passive avoidance task

This learning and memory test was performed in two chambers, which were square boxes, identical size (12×10×12 cm), juxtaposed as illuminated and dark. A lamp (50 W) was placed 1 m above one chamber for illumination. Each test involved two separate trials, a training trial and a test trial. For the training trial, the mice were initially placed in the illuminated chamber. When the mouse entered the dark chamber, an electrical shock (0.5 mA) for 3 sec was delivered through stainless steel rods. The latency times once the mice had entered the dark compartment were measured using a stopwatch. A test trial was performed 24 h following the training trial, and latency times to re-enter the dark chamber were measured up to 300 sec. FAE or vehicle were administered 40 min prior to the acquisition trials. Each acquisition trial was performed 10 min following a single scopolamine treatment (2 mg/kg, i.p.).

### Step-down test

This test was used to measure inhibitory avoidance. The apparatus comprised a plastic chamber (12×12×18 cm) with an elevated rubber platform (4.8×4.8×4.5 cm) placed on the left side wall. The floor was made of caliber stainless steel bars (0.1 cm in length) placed in parallel, 0.5 cm apart. Mice were housed in a dimly lit room for ≥30 min prior to the experiment. On the first training day, mice were exposed to a 5-min learning course, during which they were permitted to move freely throughout the chamber prior to being placed on the platform. If the animals stepped down from the platform (i.e. an error trial), they were exposed to an electric foot shock (36 V, AC). After 24 h, latency was reassessed and recorded as the learning grade (latency), which was taken as a measure of memory retention. Each acquisition trial was performed 10 min after a single scopolamine treatment (2 mg/kg, i.p.). FAE or vehicle were administered 30 min prior to the trials.

### T-maze task

This task was used to evaluate spatial memory (via spontaneous alternation behavior), as previously described by Amico *et al* (16), with slight modifications. The maze (Ugo Basile, Comerio, Italy) was made of a non-reflective base plate and plastic arms (28×5×10 cm). Training consisted of one single session, which started with one forced-choice trial, followed by 14 free-choice trials. In the forced-choice trial, either the left or right goal arm was blocked by a guillotine door. Once the animal was released from the starting arm, it was allowed to explore the maze by entering the open goal arm and returning to the start position where it was confined for 5 sec by the lowering of the guillotine door. During the 14 free-choice trials, the animal was free to choose between the left and right goal arm. Immediately upon entering one goal arm, the other goal arm closed, and once the mouse returned to the starting arm, the next free-choice trial started following a 5-sec restraint in the starting arm. Animals were removed from the maze as soon as the 14 free-choice trials were performed or 15 min had elapsed. The series of arm entries were recorded visually, and the alternations were calculated as a percentage of the actual alternations/the total possible alternations. Animals that did not complete the test within 15 min were excluded from the analyses since they were considered to exhibit poor exploratory behavior. The T-maze task was performed 40 and 20 min following FAE and scopolamine injections, respectively.

### Statistical analysis

All data are expressed as the mean ± standard error of the mean, and were analyzed using a one-way analysis of variance followed by the Tukey’s post-hoc test. P<0.05 was considered to indicate a statistically significant difference. Statistical analysis was performed using GraphPad Prism version 5 (Graph Pad Software, San Diego, CA, USA).

## Results

### T-maze and MWM task

**Figure 1:**
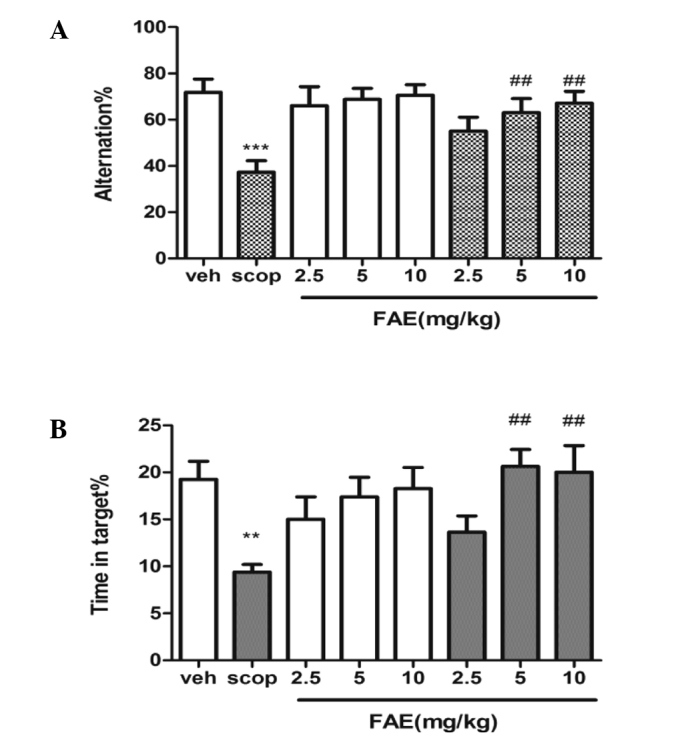
FAE rescues scopolamine-induced spatial memory deficits. The effect of FAE treatment on (A) spontaneous alternation behavior (T-maze task) and (B) MWM in a scopolamine-induced amnesia model in mice. In the T-maze, FAE [(2.5, 5 and 10 mg/kg, orally (p.o.)] was administered 20 min prior to scopolamine [10 mg/kg, intraperitoneally (i.p.)]; and in the MWM, FAE (2.5, 5 and 10 mg/kg, p.o.) was administered 20 min prior to scopolamine (2 mg/kg, i.p.). Data are expressed as mean ± standard error of the mean. Statistical analysis was performed using a one-way analysis of variance, followed by post-hoc Tukey’s test where appropriate, ^***^P<0.001 vs. controls (veh); ^**^P<0.001 vs. controls (veh); ^##^P<0.01 vs. scopolamine-treated animals (scop). FAE, Fructus Akebiae; MWM, Morris water maze.

In the T-maze task, the percentage of alternations was found to be positively correlated with the cognitive ability of the animals (Fig. 1A). Scopolamine significantly (P<0.001) reduced the percentage of alternations compared with that of the vehicle controls. However, administration of 5 and 10 mg/kg FAE markedly reversed scopolamine-induced memory deficits (P<0.01). No effect was observed in the control following FAE treatment at these two doses. In the MWM task, the time spent in the correct quadrant was significantly decreased in scopolamine-treated mice compared with that of the vehicle (control) group (P<0.001), and treatment with FAE (5 and 10 mg/kg) was found to markedly reverse this effect (P<0.01). Treatment with FAE at all three doses had no effect on the control mice.

### Novel object recognition test

**Figure 2:**
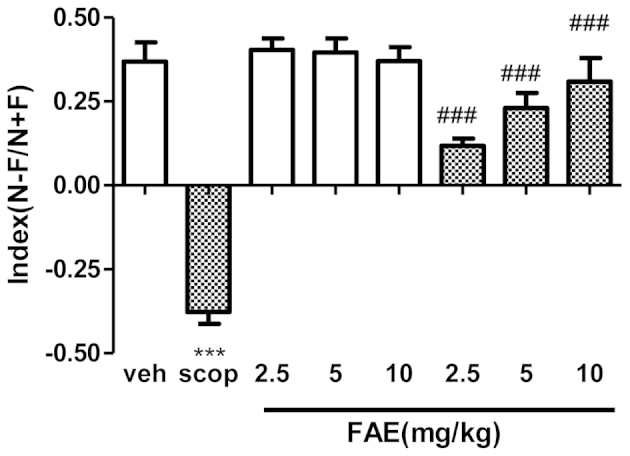
Effect of FAE treatment on the novel object recognition tasks using a scopolamine-induced amnesia model in rats. In tests, FAE (2.5, 5 and 10 mg/kg, orally) was administered 20 min prior to scopolamine (10 mg/kg, intraperitoneally) prior to the first trial only. Data are expressed as the mean ± standard error of the mean. Statistical analysis was performed using one-way analysis of variance followed by post-hoc Tukey’s test. ^***^P<0.001 vs. controls (veh); ^###^P<0.001 vs. scopolamine-treated animals (Scop). FAE, Fructus Akebiae.

During the adaptation phase to the open field box on day 1, all animals exhibited similar locomotor activity and anxiety levels. Furthermore, all rats equally explored the familiar objects during T1. Vehicle-treated animals exhibited a positive index (0.46±0.02) during T2, indicating a good recognition memory (Fig. 2). However, scopolamine-treated rats showed a significant (P<0.001) negative index (−0.41±0.12) compared with the vehicle group, indicating a poor exploration of the object types. This effect was fully (P<0.001) reversed by all doses of FAE. The discrimination index was not significantly altered in the vehicle-treated control animals following treatment with FAE.

### Passive avoidance task

**Figure 3:**
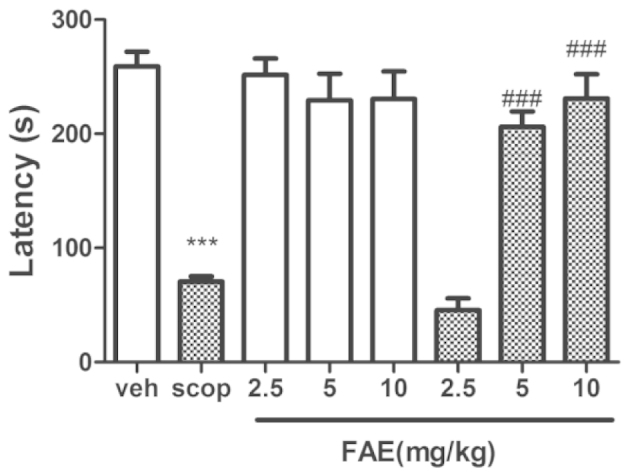
Effect of FAE treatment in the passive avoidance test using a scopolamine-induced amnesia model in mice. In tests, FAE (2.5, 5 and 10 mg/kg, orally) was administered 20 min prior to scopolamine (10 mg/kg, intraperitoneally) prior to the first trial only. Data are expressed as the mean ± standard error of the mean. Statistical analysis was performed using a one-way analysis of variance followed by post-hoc Tukey’s test. ^***^P<0.001 vs. controls (veh); ^###^P<0.001 vs. scopolamine-treated animals (scop). FAE, Fructus Akebiae.

The step-through latency of scopolamine-treated mice was significantly (P<0.001) shorter than that of vehicle-treated mice (Fig. 3). The retention latency during the pre-training phase was shortened by scopolamine treatment. This response was significantly (P<0.01) reversed by 5 and 10 mg/kg FAE.

### Step-down test

**Figure 4:**
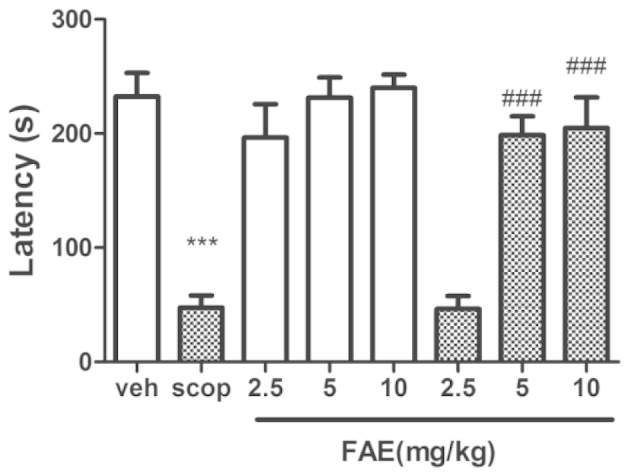
Effect of FAE treatment in the step-down test using a scopolamine-induced amnesia model in mice. In tests, FAE (2.5, 5 and 10 mg/kg, orally) was administered 20 min prior to scopolamine (10 mg/kg, intraperitoneally) prior to the first trial only. Data are expressed as the mean ± standard error of the mean. Statistical analysis was performed using one-way ANOVA followed by post-hoc Tukey’s test. ^***^P<0.001 vs. controls (veh); ^###^P<0.001 vs. scopolamine-treated animals (scop). FAE, Fructus Akebiae.

The step-down latency of scopolamine-treated mice was significantly (P<0.01) shorter compared with that of the vehicle-treated controls (Fig. 4). This effect was significantly (P<0.001) reversed by 5 and 10 mg/kg FAE.

## Discussion

Traditional Chinese herbs and other plants have been increasingly recognized as effective natural materials for the treatment of diseases and the alleviation of symptoms. Certain traditional herbs may effectively treat specific cognitive disorders, as well as manage cognitive decline during aging (17). A number of studies have demonstrated that FAE ameliorates aging-associated cognitive deterioration, cerebral ischemia and chemical injury to the brain (18,19). In the present study, the cognitive-enhancing activity of FAE in a scopolamine model of dementia in mice and rats was investigated.

Cholinergic neurons have a major role in cognitive function, and their loss from the hippocampus is a characteristic feature of AD. Scopolamine impairs learning and memory in rodents and humans. It is used to create an experimental model of memory impairment and has been extensively used to screen for drugs that have potential therapeutic effect in dementia (20,21). Scopolamine has been shown to cause reversible symptoms of senile dementia in younger individuals (22). The passive avoidance task, novel object recognition test, step-down test and MWM task are used to evaluate learning and memory impairment in animals (14,23,24).

Early symptoms of AD include deficits in short-term episodic memory, attention and spatial orientation (25,26). Episodic memory is a type of declarative memory that depends on the ability to remember in a determined temporal and spatial context (27), and is particularly vulnerable to normal aging and dementia (20). Spatial memory is a subtype of episodic memory, storing past information of events within the spatiotemporal frame (14). To evaluate the effect of FAE on short- and long-term spatial memory, the T-maze and the MWM task were used, as previously described by Gacar *et al* (14). In the two behavioral tests, scopolamine induced a marked decline in cognitive performance, and FAE (5 and 10 mg/kg) was found to significantly attenuate this effect.

To further investigate the effect of FAE on other types of memory, the novel object recognition test and the passive avoidance task were performed. The novel object recognition test evaluates recognition memory (28), whilst the passive avoidance task is dependent on the amygdala and evaluates emotional memory. Passive avoidance has been associated with long-term or reference memory and has been used to study learning and memory following a stressful stimulus (29). In these behavioral tests, treatment with FAE (5 and 10 mg/kg) was shown to prevent scopolamine-mediated cognitive impairment.

The present study clearly demonstrates that FAE significantly attenuates scopolamine-mediated cognitive impairment. In conclusion, the results suggest that FAE may be a potential novel therapeutic strategy for the treatment of dementia.
